# Suicidal Ideation and Death by Suicide as a Result of the COVID-19 Pandemic in Spanish-Speaking Countries: Systematic Review

**DOI:** 10.3390/jcm12216700

**Published:** 2023-10-24

**Authors:** Nicolás Valle-Palomino, Mirtha Mercedes Fernández-Mantilla, Danae de Lourdes Talledo-Sebedón, Olinda Victoria Guzmán-González, Vanessa Haydee Carguachinchay-Huanca, Alfonso Alejandro Sosa-Lizama, Brunella Orlandini-Valle, Óscar Manuel Vela-Miranda

**Affiliations:** 1 Escuela de Psicología, Universidad César Vallejo, Piura 20001, Peru; mfernandez@ucv.edu.pe (M.M.F.-M.); danaedelourdest@gmail.com (D.d.L.T.-S.); victoriaguzmangonzales028@gmail.com (O.V.G.-G.); vh27carguachinchay2002@gmail.com (V.H.C.-H.); alfonsososalizama@gmail.com (A.A.S.-L.); ovela@ucv.edu.pe (Ó.M.V.-M.); 2 College of Science, Technology, and Health, Lindenwood University, St. Charles, MO 63301, USA; bruorlandini012@gmail.com

**Keywords:** suicidal ideation, death by suicide, COVID-19, systematic review

## Abstract

Suicidal behaviors and constructs are putting at risk the accomplishment of Objective 3 of Agenda 2030 for sustainable development in Spanish-speaking countries. The current study’s principal objective is to explain the presence of suicidal ideation and deaths by suicide as a result of the COVID-19 pandemic in Spanish-speaking countries based on a review of the scientific literature. The PRISMA model was used as the main method while considering the criteria of periodicity, language, typology, and country in order to choose the 28 articles that were analyzed from the following three databases: SCOPUS, Web of Science, and ProQuest Coronavirus Research Database. Suicidal ideation and death by suicide exhibited a growth trend during the COVID-19 pandemic, including post-pandemic, especially in kids, adolescents, and young women of university age belonging to lower socioeconomic strata and presenting risk factors like living in rural areas, poor mental health, unemployment, and family death due to COVID-19.

## 1. Introduction

Mental health in Latin America is notably inadequate, being one of the most important difficulties that the government should deal with, in the scope of public health. The most influential mental health disorders in the general population, such as substance overdose, depression, schizophrenia, anxiety, and impulsive behaviors, are determined to be some of the main causes of mortality [1,2], and they could directly impact the sustainable development of a country, in accordance with Objective 3 of Agenda 2030 and the objectives of sustainable development [3].

This situation has notably increased after the COVID-19 pandemic, leaving lingering side effects on the emotional stability of the population and creating the need for governments to implement immediate strategies in their public health systems [4,5,6].

It is estimated that nearly one billion subjects worldwide exhibited symptoms of mental health deficits during just the first initial period of confinement due to the pandemic [7].

Out of all global deaths due to the pandemic, 32% were registered in Spanish-speaking countries, generating a highly impactful emotional overload that has contributed to the mental health deficit and with suicide as one of the most frequent responses [8]. In this context, governments, through their public health systems, have the responsibility to propose programs with coherent budgets, including the utilization of adequate technologies, that allow timely detection of suicide risk [9,10].

It is vital to specify that individuals who tested positive for COVID-19 have an approximately 50% higher probability of experiencing suicidal ideation compared to those who did not contract the virus or had mild symptoms. However, specific data does not exist yet, which is why diverse organizations are currently studying the relationship between depression and suicidal thoughts in the context of the coronavirus [11]. In other words, individuals who had experiences closely related to COVID-19 presented higher levels of depression and suicidal ideation in comparison to those who had no contact with the disease, with front-line professionals being an example of those individuals [12].

To contextualize the study, basic definitions of the terminology that will be used in the study are provided. Suicidal ideation is defined as thoughts about self-harm, with or without planning of which method to use to cause one’s own death. Suicide is the act of causing one’s own death [13].

In the year 2021, during the World Economic Forum, speakers urged the prioritization of suicide prevention, following the presentation of survey results involving participants from Chile, Brazil, Peru, and Canada, which revealed a significant decline in mental health due to COVID-19 [14]. The health system’s inefficient response to the pandemic led to an inequality in the attended population, with the poorest being at a disadvantage and currently experiencing a greater impact on their mental health [15].

Every year, around 700,000 people worldwide attempt to take their own lives. Suicide ranks as the fourth leading cause of mortality among individuals aged 15 to 29, achieving an elevated percentage (up to 77%) in countries with low to middle-income economies [16]. In Latin America, suicides are increasing annually. Suicide attempts occur 10 to 20 times more frequently than each death by suicide [17].

In the context of the pandemic, 79% of deaths by suicide were committed by males and 21% by females, with the three main causes being healthcare worker exhaustion, feelings of loneliness, and testing positive for COVID-19 [15,18]. The above sustains the increase in attended cases in health services [19].

In Peru, more than 6000 individuals have taken their own lives in the past 10 years. However, 31% solely corresponds to the years 2020, 2021, and 2022, confirming an increase during the pandemic [20]. In Mexico, the incidence of suicide among adults during the pandemic period was the highest in the last 50 years, evidencing a public health crisis [21]. The situation is no different in Argentina, where an average of eight people commit suicide daily. This country also presented a mental health deficit, which worsened progressively during the confinement period [22].

The Pan American Health Organization is focusing on addressing all the factors involved in suicide prevention in the Americas. One of the sustainable development goals is to reduce the suicide mortality rate by one-third by the year 2030. This objective is directly aligned with the current strategic plan proposed for the years 2020–2025, which includes suicide as one of its principal indicators to work on [14]. Suicide prevention is a global necessity that should be oriented toward the general population to enhance mental health by applying effective and feasible methods to different contexts at the individual level, community level, and social level [23].

The COVID-19 pandemic has not affected as many people as other challenges that humanity has faced; it is also not the most lethal. However, today’s world, due to globalization, took a spectator stance, thinking it had everything under control, which limited its responsiveness. The velocity of spread of COVID-19 broke the sense of security that the world had, with this pandemic with unique characteristics [24,25,26] affecting people’s resources [15].

A pandemic is understood as an epidemic that has spread across several countries, continents, or the whole world, affecting a great number of people [14]. The COVID-19 pandemic presented alarming levels of spread and severity of the coronavirus disease (COVID-19), which is a disease caused by the SARS-CoV-2 virus [16].

Studies exist which cover the proposed topic that, for the most part, are conducted by epidemiologists and show the statistics for the number of cases, frequencies, etc. Thus, it is necessary to continue investigating to complete the panorama [27].

Based on the previously presented information, we can confirm that despite multiple research studies and literature reviews regarding suicidal ideation and deaths by suicide in the context of the pandemic, it is evident that there is a deficit in analysis and meticulous synthesis. This justifies the current proposal, which will provide a comprehensive overview specifically focusing on Latin America.

The question that guided this study was: How do suicidal ideation and death by suicide present themselves as a result of the COVID-19 pandemic in Spanish-speaking countries, based on a review of the scientific literature?

The principal objective is to explain the presence of suicidal ideation and deaths by suicide due to the COVID-19 pandemic in Spanish-speaking countries, based on a review of scientific literature.

The specific objectives are:

- Selecting the scientific evidence on suicidal ideation and death by suicide resulting from the COVID-19 pandemic in Spanish-speaking countries;
- Analyzing the scientific evidence on suicidal ideation and death by suicide resulting from the COVID-19 pandemic in Spanish-speaking countries.

## 2. Method

The current study was developed based on the elements for structuring systematic reviews as per the 2020 PRISMA (Preferred Reporting Items for Systematic Reviews and Meta-Analyses) statement, which is also designed to assist authors in adequately documenting their work [28,29].

### 2.1. Eligibility Criteria for Articles

The research studies were selected are based on the following criteria:

- Study period: January 2021–May 2023;
- Article language: Spanish or English;
- Language of the country where the study was conducted: Spanish;
- Article type: original;
- Open access article;
- Countries where the study was conducted: Spain, Mexico, Colombia, Argentina, Chile, and Peru;
- The sample revealed suicidal ideation and death by suicide between January 2021 and May 2023, the period in which the World Health Organization declared the end of the health emergency.

### 2.2. Sources of Information, Search Strategy, Study Selection Process, and Data Selection Process

Three scientific databases were utilized to retrieve the selected published articles: SCOPUS, Web of Science, and ProQuest Coronavirus Research Database. The database search was conducted in June and July of 2023. Queries were formulated by combining three main keywords, (1) suicidal ideation, (2) death by suicide, and (3) COVID-19, and included translations in the English language.

The search strategy used in this study is presented in Table 1.

For the study selection, an initial review was conducted based on the title, abstract, keywords, and country. Articles that passed the initial review were read in-depth to decide whether they would continue for further review or be discarded. In this second phase, the article’s title, bibliographic reference, year of publication, publishing journal, DOI, indexing database, article type, country where the study was conducted, objective, method, results, and conclusions were recorded. This was performed using a matrix in Microsoft Excel.

The selection process of the studies was conducted by three independent reviewers, and any discrepancies were resolved through discussion and, if necessary, by voting, in which the majority vote determined whether a study was included or discarded among the selected articles.

### 2.3. Data Analysis

Through the course of the current research study, it was noted that a repetitive trend of elements was related to the research objectives. These elements are: (1) age, (2) gender, (3) socioeconomic level, and (4) risk factors such as living in rural areas, unemployment, and family death due to COVID-19. These have also been taken into consideration to organize the results, corresponding analysis, and discussion.

## 3. Results

**Figure 1:**
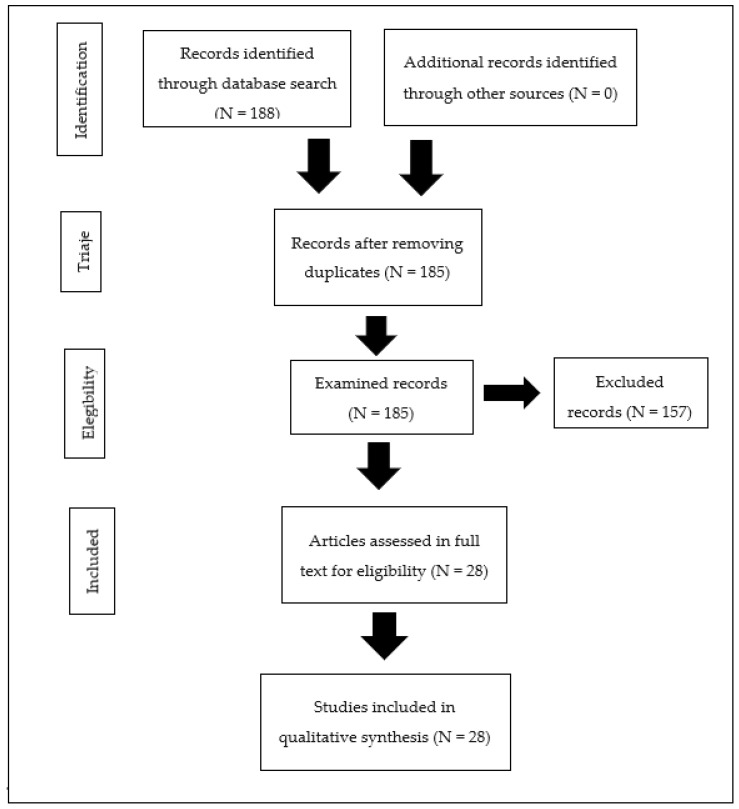
Selection process flowchart.

Following the application of the search strategies, 6295 initial references were obtained. Subsequently, by applying the selection criteria, 188 references were obtained. During the study selection process, 3 duplicate references were identified, meaning they were identical. Additionally, 157 references that did not align with the inclusion criteria set in the initial review (title, abstract, keywords, and study country) were eliminated (Figure 1). Ultimately, 28 references were included at the conclusion of the selection process (Table 2).

## 4. Discussion

The principal objective of the current study was to explain the presence of suicidal ideation and death by suicide due to the COVID-19 pandemic in Spanish-speaking countries. After conducting the search in SCOPUS, Web of Science, and ProQuest Coronavirus Research Database, 28 references were identified, demonstrating a representation of what is happening with suicidal ideation and death by suicide post-pandemic in Spanish-speaking countries.

The second specific objective was to analyze scientific data on suicidal ideation and death by suicide as a result of the COVID-19 pandemic in Spanish-speaking countries, finding four elements that allow the realization of said analysis: (a) age, (b) gender, (c) socioeconomic level, and (d) risk factors such as living in rural areas, unemployment, and family death due to COVID-19; these elements were observed with a greater recurrence upon reviewing the references.

(a) Age:

Based on observations from the publications, the most vulnerable age groups with a higher tendency towards suicidal behaviors and constructs are children and adolescents, who must face new demands for development and well-being. These new demands can lead to high-risk constructs related to the severity of depressive and anxiety disorders, emphasizing the importance of implementing multidisciplinary prevention strategies. It should be noted that this issue has developed progressively in tandem with the increase in COVID-19 cases [30,33,45].

On the other hand, it was identified that suicidal ideation in university students, women, and adolescents younger than 20 years old as a result of the uncertainty generated by COVID-19, demonstrating a relationship between the increase in stress and poor mental health, causing an increase in suicidal behaviors [43,47].

(b) Gender:

Following the onset of the pandemic, there was an alarming increase in cases of suicidal behaviors and constructs in healthcare centers, which continued throughout the entire health emergency, with a higher incidence in women [19,36].

During the pandemic, within the healthcare workforce, women exhibited a higher tendency towards suicidal behaviors [39].

(c) Socioeconomic level:

During the health emergency, the lower economic sectors suffered great losses in employment and from economic limitations, which can be related to mental health difficulties such as anxiety disorders, depressive disorders, etc., that are identified as factors causing suicidal behaviors and constructs [40].

The research studies demonstrated an increased tendency to suicidal behaviors and constructs in the subsequent years after the beginning of the pandemic, which continues to increase, with a noticeably higher incidence in populations with lower socioeconomic levels and among young women [36].

It should be emphasized that although suicidal ideation was observed in different sociodemographic strata, a higher incidence was observed among healthcare personnel [32].

(d) Risk factors such as living in rural areas, unemployment, and family death due to COVID-19:

During the health emergency, the risk factors with the highest incidence were diagnosis of depression and anxiety, violence, substance abuse, perceived low income, living in rural areas, unemployment, suspicion or diagnosis of COVID-19, and the death of a family member due to COVID-19 [35,42,52,53,57,58]. Other risk factors leading to depressive symptoms with a tendency towards suicidal behaviors include marital status, not having completed regular basic education, leading a sedentary lifestyle, and physical distancing [50,55]. These risk factors contributed to highly significant suicidal behaviors and constructs [37,41,49,59,60,61].

The four elements mentioned previously correlate with what is currently happening in the Spanish-speaking countries included in this research study; the national statistical institutes have observed an increase in suicidal behaviors and constructs.

In Spain, in the year 2020, there were 3941 suicides recorded, while in the year 2022, that number had a growth rate of 90.9% [62]. In Mexico, in the year 2020, 7896 suicides were recorded; the figure increased to 8432 recorded in the year 2021. While it slightly decreased for the year 2022, 8237 cases were recorded [63].

In Colombia, in the year 2020, 2748 deaths by suicide were reported, while in the year 2022, it was listed that 2385 people ended their lives [64]. For the year 2019, pre-pandemic, in Argentina, there were 3300 suicides. According to the 2022 census, post-COVID-19 pandemic, figures of 4000 suicides per year have been reached [65].

In Chile, accurate yearly statistics regarding the number of suicides in the country since the beginning of the pandemic have not been successfully identified. However, some authors mentioned that in the year 2020, at the beginning of the health emergency, there was a decrease in suicide cases in relation to pre-pandemic figures. For the year 2021, a substantial increase was observed and has been maintained after the pandemic concluded [66].

In Peru, in the year 2020, 569 suicide cases were reported. For the year 2021, 521 cases were reported, and up until October, 612 cases were recorded [17].

The statistics from the presented countries show a trend of growth in the total number of cases; this is supported by the fact that suicidal constructs exhibited a significant increase throughout the development of the COVID-19 pandemic, with expectations of it persisting long term [56].

In this sense, actions of prevention and intervention deserve a special analysis beyond the knowledge base that the scientific investigation is providing.

The limitations identified for the current study are (a) the use of three databases of high impact and prioritizing among the selection criteria the open access filter. Although the investigators have the power to choose their criteria and resources, it would be important to develop new investigations that include the spectrum of open access and closed access to accomplish a more integrative view. (b) Nowadays, knowledge is updated quickly and constantly, so the strategies utilized to identify the research articles could be outdated in a short amount of time, due to the progress of information.

According to the conducted analysis and in coherence with the results of the reviewed scientific publications, it can be confirmed that the prevention of psychological constructs and suicidal behaviors subsequent to the pandemic is a fundamental element that governments should address in all Spanish-speaking countries, within the framework of Objective 3 of Agenda 2030 for sustainable development [50].

It is vital to propose policies and programs that define long-term prevention strategies to address suicidal ideation and potential self-harming actions that can lead to traumatic experiences within families, especially among members with risk factors [31,34,54,67,68]. The healthcare sector, having been on the frontline, merits special attention in the prevention efforts [46,69].

It is important for Hispanic American countries to identify the groups with higher suicide tendencies based on the research about post-pandemic effects, as the COVID-19 pandemic has contributed to a decrease in mental health within the population [38,44].

The individuals that comprise diverse social groups (the armed forces, the academic community, civil society, and others) have the responsibility to educate themselves on topics of suicide constructs as a product of the pandemic, to contribute to the efforts that governmental organizations may undertake [48,51,70].

## 5. Conclusions

Suicidal ideation and death by suicide exhibited a growth trend during the COVID-19 pandemic, including post-pandemic, especially in kids, adolescents, and young women of university age belonging to lower socioeconomic strata and presenting risk factors such as living in rural areas, poor mental health, unemployment, and family death due to COVID-19.

The growth trend of suicidal ideation and death by suicide becomes a limiting factor for achieving Objective 3 of Agenda 2030 for sustainable development in Spanish-speaking countries.

Governments must propose policies and programs that enable the development of long-term prevention strategies and address the consequences of the increase in post-pandemic suicidal ideation and deaths by suicide.

Among the identified limitations of the current study is the utilization of an open access criterion as one of the selection criteria, and the constant updating of information that renders information search strategies outdated.

## Figures and Tables

**Table 1:** Search strategy.

No.	Database	Search Strategy
01	SCOPUS	(TITLE-ABS-KEY (SUICIDAL AND IDEATION) AND TITLE-ABS-KEY (COVID)) AND (LIMIT-TO (PUBSTAGE, “FINAL”)) AND (LIMIT-TO (OA, “ALL”)) AND (LIMIT-TO (AFFILCOUNTRY, “SPAIN”) OR LIMIT-TO (AFFILCOUNTRY, “MEXICO”) OR LIMIT-TO (AFFILCOUNTRY, “ARGENTINA”) OR LIMIT-TO (AFFILCOUNTRY, “PERU”) OR LIMIT-TO (AFFILCOUNTRY, “COLOMBIA”) OR LIMIT-TO (AFFILCOUNTRY, “CHILE”)) AND (LIMIT-TO (PUBYEAR, 2023) OR LIMIT-TO (PUBYEAR, 2022) OR LIMIT-TO (PUBYEAR, 2021) OR LIMIT-TO (PUBYEAR, 2020)) AND (LIMIT-TO (DOCTYPE, “AR”)) AND (LIMIT-TO (SUBJAREA, “MEDI”) OR LIMIT-TO (SUBJAREA, “PSYC”)) AND (LIMIT-TO (EXACT KEYWORD, “SUICIDAL IDEATION”) OR LIMIT-TO (EXACT KEYWORD, “COVID-19”) OR LIMIT-TO (EXACT KEYWORD, “PANDEMIC”)) AND (LIMIT-TO (LANGUAGE, “ENGLISH”) OR LIMIT-TO (LANGUAGE, “SPANISH”))
02	Web of Science	Results for SUICIDAL IDEATION (All Fields) AND COVID (All Fields) and Open Access and 6.24 Psychiatry & Psychology or 1.21 Psychiatry (Citation Topics Meso) and 1.21.430 Suicide (Citation Topics Micro) and 2020 or 2023 or 2021 or 2022 (Publication Years) and Article or Review Article (Document Types) and Psychiatry or Psychology Multidisciplinary or Psychology Clinical or Medicine General Internal or Psychology (Web of Science Categories) and All Open Access (Open Access) and SPAIN or ARGENTINA or COLOMBIA or MEXICO or PERU (Countries/Regions) and English or Spanish (Languages)
03	ProQuest Coronavirus Research Database	Searched for: (SUICIDAL IDEATION AND COVID) AND (location.exact (“Spain” OR “Mexico” OR “Peru” OR “Chile” OR “Colombia” OR “Argentina”) AND at.exact (“Article”) AND subt.exact (“COVID-19” OR “suicides & suicide attempts”) AND la. Exact (“ENG”) AND PEER (yes)) Database: Coronavirus Research Database Results: 123

**Table 2:** Included articles.

No.	Excel Matrix Code	Title	DOI
1	1	Dramatic increase of suicidality in children and adolescents after COVID-19 pandemic start: A two-year longitudinal study [30]	https://doi.org/10.1016/j.jpsychires.2023.04.014
2	4	Suicide attempt before and during the COVID-19 pandemic: A comparative study from the emergency department [19]	https://doi.org/10.1016/j.semerg.2023.101922
3	6	COVID-19 Pandemic Control Measures and Their Impact on University Students and Family Members in a Central Region of Spain [31]	https://doi.org/10.3390/ijerph20054470
4	7	Suicidal thoughts and burnout among physicians during the first wave of the COVID-19 pandemic in Spain [32]	https://doi.org/10.1016/j.psychres.2023.115057
5	8	COVID-19 Pandemic Has Changed the Psychiatric Profile of Adolescents Attempting Suicide: A Cross-Sectional Comparison [33]	https://doi.org/10.3390/ijerph20042952
6	10	Prevalence of suicidal behavior in a northeastern Mexican border population during the COVID-19 pandemic [34]	https://doi.org/10.3389/fpsyg.2022.984374
7	12	Social determinants associated with suicidal ideation during the COVID-19 pandemic in Mexico [35]	https://doi.org/10.21149/13744
8	14	Impact of the COVID-19 Pandemic on the Incidence of Suicidal Behaviors: A Retrospective Analysis of Integrated Electronic Health Records in a Population of 7.5 Million [36]	https://doi.org/10.3390/ijerph192114364
9	17	Psychological Impact and Risk of Suicide in Hospitalized COVID-19 Patients, During the Initial Stage of the Pandemic: A Cross-Sectional Study [37]	https://doi.org/10.1097/PTS.0000000000000974
10	21	Increased incidence of high-lethality suicide attempts after the declaration of the state of alarm due to the COVID-19 pandemic in Salamanca: A real-world observational study [38]	https://doi.org/0.1016/j.psychres.2022.114578
11	22	Four-month incidence of suicidal thoughts and behaviors among healthcare workers after the first wave of the Spain COVID-19 pandemic [39]	https://doi.org/10.1016/j.jpsychires.2022.02.009
12	25	The Mental Health of Employees with Job Loss and Income Loss during the COVID-19 Pandemic: The Mediating Role of Perceived Financial Stress [40]	https://doi.org/10.3390/ijerph19063158
13	29	Comparison of suicide attempts among nationally representative samples of Mexican adolescents 12 months before and after the outbreak of the COVID-19 pandemic [41]	https://doi.org/10.1016/j.jad.2021.10.111
14	31	Anxiety and Fear of COVID-19 among Shantytown Dwellers in The Megacity of Lima [42]	https://doi.org/10.2174/18749445-v15-e221026-2022-69
15	34	Influence of COVID-19 pandemic uncertainty in negative emotional states and resilience as mediators against suicide ideation, drug addiction and alcoholism [43]	https://doi.org/10.3390/ijerph182412891
16	39	Impact of COVID-19 pandemic outbreak on mental health of the hospital front-line healthcare workers in Chile: a difference-in-differences approach [44]	https://doi.org/10.1093/pubmed/fdac008
17	42	Psychosocial correlates of suicidal behavior among adolescents under confinement due to the COVID-19 pandemic in Aguascalientes, Mexico: A cross-sectional population survey [45]	https://doi.org/10.3390/ijerph18094977
18	43	Thirty-day suicidal thoughts and behaviors among hospital workers during the first wave of the Spain COVID-19 outbreak [46]	https://doi.org/10.1002/da.23129
19	47	Suicidal ideation, anxiety, social capital, and sleep among Colombians in the first month of COVID-19-related physical isolation [47]	https://doi.org/10.17081/psico.24.45.4075
20	48	Current impact and future consequences of the pandemic on children’s and adolescents’ health [48]	http://dx.doi.org/10.5546/aap.2021.eng.e594
21	50	Thirty-day suicidal thoughts and behaviours in the Spanish adult general population during the first wave of the Spain COVID-19 pandemic [49]	https://doi.org/10.1017/S2045796021000093
22	2	Influence of Loneliness, Anxiety, and Depression on Suicidal Ideation in Peruvian Adults during the COVID-19 Pandemic [50]	https://doi.org/10.3390/su15043197
23	3	Predictive Factors of Suicidal Ideation in Spanish University Students: A Health, Preventive, Social, and Cultural Approach [51]	https://doi.org/10.3390/jcm12031207
24	7	Suicide Risk in Military Personnel during the COVID-19 Health Emergency in a Peruvian Region: A Cross-Sectional Study [52]	https://doi.org/10.3390/ijerph192013502
25	24	COVID-19 Fear, Resilience, Social Support, Anxiety, and Suicide among College Students in Spain [53]	https://doi.org/10.3390/ijerph18158156
26	42	How is COVID-19 affecting patients with obsessive–compulsive disorder? A longitudinal study on the initial phase of the pandemic in a Spanish cohort [54]	https://doi.org/10.1192/j.eurpsy.2021.2214
27	53	Health Plans for Suicide Prevention in Spain: A Descriptive Analysis of the Published Documents [55]	https://doi.org/10.3390/nursrep12010009
28	54	Changes in psychiatric emergencies during COVID-19 pandemic lockdown in El Bierzo (Spain) [56]	https://doi.org/10.1192/j.eurpsy.2022.658

## Data Availability

The data that support the findings of this study are available on request from the corresponding author (N.V.-P.).

## References

[1] Mejía-Zambrano H., Ramos-Calsín L. (2022). Prevalencia de los principales trastornos mentales durante la pandemia por COVID-19. Rev. Neuro-Psiquiatr..

[2] Navarro-Gómez N. (2017). El suicidio en jóvenes en España: Cifras y posibles causas. Análisis de los últimos datos disponibles. Clínica Y Salud.

[3] Naciones Unidas La Agenda 2030 y los Objetivos de Desarrollo Sostenible: Una Oportunidad Para América Latina y El Caribe (LC/G.2681-P/Rev.3). https://www.un.org/sustainabledevelopment/es/development-agenda/.

[4] Bustamante F., Urquidi C., Florenzano R., Barrueto C., Hoyos J., Ampuero K., Terán L., Figueroa M.I., Farías M., Rueda M.L. (2018). El programa RADAR para la prevención del suicidio en adolescentes de la región de Aysén, Chile: Resultados preliminares. Rev. Chil. Pediatría.

[5] Dana A. (2023). The engaged community action for preventing suicide (ECAPS) model in Latin America: Development of the ¡PEDIR! program. Soc. Psychiatr. Psychiatr. Epidemiol..

[6] Institutional Repository for Information Sharing. https://iris.paho.org/handle/10665.2/57504.

[7] Salinas-Rodríguez A., Argumedo G., Hernández-Alcaraz C., Contreras-Manzano A., Jáuregui A. (2023). Depression, Anxiety, and Stress Scale: Factor validation during the first COVID-19 lockdown in Mexico. Rev. Latinoam. Psicol..

[8] Voz de América. https://www.vozdeamerica.com/a/la-ops-advierte-grave-deterioro-de-salud-mental-en-latinoamerica-y-apuntala-plan-para-hacer-frente-al-problema/7130756.html.

[9] Arilla-Andrés S., García-Martínez C., Lopez-Del Hoyo Y. (2022). Detección del riesgo de suicidio a través de las redes sociales. Int. Technol. Sci. Soc. Rev..

[10] Serrani D. (2017). Psychometric validation of the Columbia-Suicide Severity rating scale in Spanish-speaking adolescents. Colomb. Médica.

[11] El Financiero. https://www.elfinanciero.com.mx/salud/2022/09/09/las-personas-que-han-padecido-covid-son-mas-vulnerables-al-suicidio-esto-revela-un-estudio/.

[12] Urdiales R., Sánchez N. (2021). Sintomatología depresiva e ideación suicida como consecuencia de la pandemia por la COVID-19. Escr. Psicol..

[13] Asociación Americana de Psicología (2013). Manual Diagnóstico y Estadístico de Trastornos Mentales.

[14] Organización Panamericana de la Salud. https://www.paho.org/es/temas/prevencion-suicidio.

[15] Bouza E., Arango C., Moreno C., Gracia D., Martín M., Pérez V., Lázaro L., Ferre F., Salazar G., Tejerina-Picado F. (2023). Impact of the COVID-19 pandemic on the mental health of the general population and health care workers. Rev. Española De Quimioter..

[16] Organización Mundial de la Salud. https://www.who.int/es/news-room/fact-sheets/detail/suicide.

[17] Bevilacqua P. (2021). Depresión y Riesgo de Suicidio en Trabajadoras Sexuales. Gac. Médica Boliv..

[18] Kumar L., George R., Mohanan M. (2022). Background of suicide amidst COVID-19 pandemic in India: A review of published literature. Ann. Indian Psychiatry.

[19] Guil J. (2023). Suicide Attempt Before and During the COVID-19 Pandemic: An Emergency Department Comparative Study. Semergen.

[20] El Comercio. https://elcomercio.pe/peru/mas-de-6-mil-peruanos-fallecieron-por-suicidio-durante-los-ultimos-10-anos-prevencion-del-suicidio-posvencion-peru-ayuda-ecdata-noticia/.

[21] El Heraldo. https://heraldodemexico.com.mx/mundo/2023/6/16/durante-la-pandemia-de-covid-19-se-incrementaron-los-suicidios-en-jovenes-514501.html.

[22] Infobae. https://www.infobae.com/salud/2023/05/05/vigilancia-epidemiologica-del-suicidio-adicciones-y-asistencia-post-covid-15-claves-del-plan-de-salud-mental-en-argentina/.

[23] Suárez Y. (2023). Estrategias para la prevención del suicidio. Med. UPB.

[24] Culebras J., San Mauro M., Vicente-Vacas L. (2020). COVID-19 y otras pandemias. J. Negat. No Posit. Results.

[25] Mukhtar F., Candilis P. (2022). Pandemics and Suicide Risk Lessons From COVID and Its Predecessors. Home Med. J. Nerv. Ment. Dis..

[26] Giner L., Vera-Varela C., de la Vega D., Zelada G., Guija J. (2022). Suicidal Behavior in the First Wave of the COVID-19 Pandemic. Springer Nat..

[27] Cañón S., Carmona J. (2018). Ideación y conductas suicidas en adolescentes y jóvenes. Rev. Pediatr. Aten Primaria.

[28] Page M.J., McKenzie J.E., Bossuyt P.M., Boutron I., Hoffmann T.C., Mulrow C.D., Shamseer L., Tetzlaff J.M., Akl E.A., Brennan S.E. (2021). Declaración PRISMA 2020: Una guía actualizada para la publicación de revisiones sistemáticas. Rev. Española Cardiol..

[29] Urrútia G., Bonfill X. (2010). Declaración PRISMA: Una propuesta para mejorar la publicación de revisiones sistemáticas y metaanálisis [PRISMA declaration: A proposal to improve the publication of systematic reviews and meta-analyses]. Med. Clin..

[30] García-Fernández L., Romero V., Izquierdo-Izquierdo M., Rodriguez V., Álvarez-Mon M., Lahera G., Santos J.L., Rodriguez-Jimenez R. (2023). Dramatic increase of suicidality in children and adolescents after COVID-19 pandemic start: A two-year longitudinal study. J. Psychiatr. Res..

[31] Pérez-Pérez L., Cárdaba-García I., Madrigal-Fernández M., Montero F., Sobas E.M., Soto-Cámara R. (2023). COVID-19 Pandemic Control Measures and Their Impact on University Students and Family Members in a Central Region of Spain. Int. J. Environ. Res. Public Health.

[32] Sánchez D.d.l.V., Irigoyen-Otiñano M., Carballo J.J., Guija J.A., Giner L. (2023). Suicidal thoughts and burnout among physicians during the first wave of the COVID-19 pandemic in Spain. Psychiatry Res..

[33] García-Liso R., Portella M., Puntí-Vidal J., Pujals-Altés E., Torralbas-Ortega J., Llorens M., Pamias M., Fradera-Jiménez M., Montalvo-Aguirrezabala I., Palao D.J. (2023). COVID-19 Pandemic Has Changed the Psychiatric Profile of Adolescents Attempting Suicide: A Cross-Sectional Comparison. Int. J. Environ. Res. Public Health.

[34] Villareal K., Peña F., Zamora B., Vargas C., Hernández I., Landero C. (2023). Prevalence of suicidal behavior in a northeastern Mexican border population during the COVID-19 pandemic. Front. Psychol..

[35] Gómez-García J., Rivera-Rivera L., Astudillo-García C., Castillo-Castillo L., Morales-Chainé S., Tejadilla-Orozco D. (2023). Social determinants associated with suicidal ideation during the COVID-19 pandemic in Mexico. Salud Pública México.

[36] Valero-Bover D., Frader M., Carot-Sans G., Parra I., Piera-Jiménez J., Pontes C., Palao D. (2022). Impact of the COVID-19 Pandemic on the Incidence of Suicidal Behaviors: A Retrospective Analysis of Integrated Electronic Health Records in a Population of 7.5 Million. Int. J. Environ. Res. Public Health.

[37] Benavente-Fernández A., Gutiérrez-Rojas L., Torres-Parejo Ú., Morón A.I.P., Ontiveros S.F., García D.V., González-Domenech P., Ramos-Bossini A.J.L. (2022). Psychological Impact and Risk of Suicide in Hospitalized COVID-19 Patients, During the Initial Stage of the Pandemic: A Cross-Sectional Study. J. Patient Saf..

[38] García-Ullán L., de la Iglesia-Larrad J.I., Remón-Gallo D., Casado-Espada N.M., Gamonal-Limcaoco S., Lozano M.T., Aguilar L., Roncero C. (2022). Increased incidence of high-lethality suicide attempts after the declaration of the state of alarm due to the COVID-19 pandemic in Salamanca: A real-world observational study. Psychiatry Res..

[39] Mortier P., Vilagut G., Alayo I., Ferrer M., Amigo F., Aragonès E., Aragón-Peña A., del Barco A.A., Campos M., Espuga M. (2022). Four-month incidence of suicidal thoughts and behaviors among healthcare workers after the first wave of the Spain COVID-19 pandemic. J. Psychiatr. Res..

[40] De Miquel C., Domènech-Abella J., Felez-Nobrega M., Cristóbal-Narváez P., Mortier P., Vilagut G., Alonso J., Olaya B., Haro J.M. (2022). The Mental Health of Employees with Job Loss and Income Loss during the COVID-19 Pandemic: The Mediating Role of Perceived Financial Stress. J. Environ. Res..

[41] Valdez-Santiago R., Valdez-Santiago R., Villalobos A., Villalobos A., Arenas-Monreal L., Arenas-Monreal L., González-Forteza C., González-Forteza C., Hermosillo-De-La-Torre A.E., Hermosillo-De-La-Torre A.E. (2022). Comparison of suicide attempts among nationally representative samples of Mexican adolescents 12 months before and after the outbreak of the COVID-19 pandemic. J. Affect. Disord..

[42] Sotomayor-Beltran C., Perez-Siguas R., Matta-Solis H., Jimenez A.P., Matta-Perez H. (2022). Anxiety and fear of COVID-19 among shantytown dwellers in the megacity of lima. Open Public Health J..

[43] García-Rivera B.R., García-Alcaraz J.L., Mendoza-Martínez I.A., Olguin-Tiznado J.E., García-Alcaráz P., Aranibar M.F., Camargo-Wilson C. (2021). Influence of COVID-19 Pandemic Uncertainty in Negative Emotional States and Resilience as Mediators against Suicide Ideation, Drug Addiction and Alcoholism. Int. J. Environ. Res. Public Health.

[44] Olivares-Tirado P., Zanga-Pizarro R. (2022). Impact of COVID-19 pandemic outbreak on mental health of the hospital front-line healthcare workers in Chile: A difference-in-differences approach. J. Public Health.

[45] Hermosillo-de-la-Torre A., Arteaga-de-la-Luna S., Acevedo-Rojas D., Juárez-Loya A., Jiménez-Tapia J.A., Pedroza-Cabrera F.J., González-Forteza C., Cano M., Wagner F.A. (2021). Psychosocial Correlates of Suicidal Behavior among Adolescents under Confinement Due to the COVID-19 Pandemic in Aguascalientes, Mexico: A Cross-Sectional Population Survey. Int. J. Environ. Res. Public Health.

[46] Mortier P., Vilagut G., Ferrer M., Serra C., Molina J.D., López-Fresneña N., Puig T., Pelayo-Terán J.M., Pijoan J.I., Emparanza J.I. (2021). Thirty-day suicidal thoughts and behaviors among hospital workers during the first wave of the Spain COVID-19 outbreak. Psicogente.

[47] Rodríguez U., León Z., Ceballos G. (2021). Suicidal ideation, anxiety, social capital, and sleep among colombians in the first month of COVID-19-related physical isolation. Psicogente.

[48] Cacchiarelli N., Eymann A., Ferraris J. (2021). Current impact and future consequences of the pandemic on children’s and adolescents’ health. Arch. Argent. Pediatr..

[49] Mortier P., Vilagut G., Ferrer M., Alayo I., Bruffaerts R., Cristóbal-Narváez P., del Cura-González I., Domènech-Abella J., Felez-Nobrega M., Olaya B. (2021). Thirty-day suicidal thoughts and behaviours in the Spanish adult general population during the first wave of the Spain COVID-19 pandemic. Epidemiol. Psychiatr. Sci..

[50] De La Cruz-Valdiviano C., Bazán-Ramírez A., Henostroza-Mota C., Cossío-Reynaga M., Torres-Prado R.Y. (2023). Influence of loneliness, anxiety, and depression on suicidal ideation in peruvian adults during the COVID-19 pandemic. Sustainability.

[51] Lázaro-Pérez C., Gómez P., Martínez-López J., Gómez-Galán J. (2023). Predictive factors of suicidal ideation in spanish university students: A health, preventive, social, and cultural approach. J. Clin. Med..

[52] Valladares-Garrido M.J., Picón-Reátegui C.K., Zila-Velasque J.P., Grados-Espinoza P., Hinostroza-Zarate C.M., Failoc-Rojas V.E., Pereira-Victorio C.J. (2022). Suicide risk in military personnel during the COVID-19 health emergency in a peruvian region: A cross-sectional study. Int. J. Environ. Res. Public Health.

[53] Muyor-Rodríguez J., Caravaca-Sánchez F., Fernández-Prados J.S. (2021). COVID-19 Fear, Resilience, Social Support, Anxiety, and Suicide among College Students in Spain. Int. J. Environ. Res. Public Health.

[54] Alonso P., Bertolín S., Segalàs J., Tubío-Fungueiriño M., Real E., Mar-Barrutia L., Fernández-Prieto M., Carvalho S.R., Carracedo A., Menchón J. (2021). How is COVID-19 affecting patients with obsessive-compulsive disorder? A longitudinal study on the initial phase of the pandemic in a Spanish cohort. Eur. Psychiatry.

[55] Sufrate-Sorzano T., Jiménez-Ramón E., Garrote-Cámara M., Gea-Caballero V., Durante A., Júarez-Vela R., Santolalla-Arnedo I. (2022). Health Plans for Suicide Prevention in Spain: A Descriptive Analysis of the Published Documents. Nurs. Rep..

[56] Zapico Y., Pelayo J., Vega S., Garcia M., Landera R., Espandian A. (2022). Changes in psychiatric emergencies during COVID-19 pandemic lockdown in El Bierzo (Spain). Eur. Psychiatry.

[57] De Macêdo D., Costa A., Karina R., Ribeiro A., Leite E., Tolstenko L. (2022). Suicidal behavior during the COVID-19 pandemic: Clinical aspects and associated factors. Acta Paul. Enferm..

[58] Efstathiou V., Stefanou M., Siafakas N., Makris M., Tsivgoulis G., Zoumpourlis V., Spandidos D., Smyrnis N., Rizos E. (2022). Suicidality and COVID-19: Suicidal ideation, suicidal behaviors and completed suicides amidst the COVID-19 pandemic (Review). Exp. Ther. Med..

[59] Irigoyen-Otiñano M., Nicolau-Subires E., González-Pinto A., Adrados-Pérez M., Buil-Reiné E., Ibarra-Pertusa L., Albert-Porcar C., Arenas-Pijoan L., Sánchez-Cazalilla M., Torterolo G. (2023). Characteristics of patients treated for suicidal behavior during the pandemic in a psychiatric emergency service in a Spanish province. Rev. Psiquiatr. Salud Ment..

[60] Pathirathna M., Nandasena H., Atapattu A., Weerasekara I. (2022). Impact of the COVID-19 pandemic on suicidal attempts and death rates: A systematic review. BMC Psychiatry.

[61] Leaune E., Samuel M., Oh H., Poulet E., Brunelin J. (2020). Suicidal behaviors and ideation during emerging viral disease outbreaks before the COVID-19 pandemic: A systematic rapid review. Prev. Med..

[62] Instituto Nacional de Estadística. https://www.epdata.es/datos/cifras-suicidio-espana-datos-estadisticas/607.

[63] Instituto Nacional de Estadística y Geografía. https://www.inegi.org.mx/app/indicadores/?ind=6200240526&tm=6#D6200240526#D6200240338.

[64] Departamento Administrativo Nacional de Estadística. https://www.dane.gov.co/.

[65] Instituto Nacional de Estadística y Censos. https://www.indec.gob.ar/.

[66] Duarte D. (2022). Suicidio e intentos de suicidio en los primeros 24 meses de pandemia por COVID-19 en Chile. Rev. Chil. Atención Primaria Y Salud Fam..

[67] Vázquez P., Armero P., Martínez-Sánchez L., García J., Bonet C., Notario F., Sánchez A., Rodríguez P., Díez A. (2023). Self-harm and suicidal behavior in children and young people: Learning from the pandemic. An. Pediatría.

[68] Mora R., María Del Carmen L. (2022). Health problems in children and adolescents during the COVID-19 pandemic. Rev. Cuba. Pediatría.

[69] Farooq S., Tunmore J., Ali W., Ayub M. (2021). Suicide, self-harm and suicidal ideation during COVID-19: A systematic review. Psychiatry Res..

[70] Zalsman G., Stanley B., Szanto K., Clarke D., Carli V., Mehlum L. (2020). Suicide in the Time of COVID-19: Review and Recommendations. Acad. Int. Investig. Suicidio (IASR).

